# Harbingers of bloom: Identifying the molecular mechanisms controlling fruit bloom formation in cucumbers

**DOI:** 10.1093/plcell/koaf187

**Published:** 2025-07-28

**Authors:** Róisín Fattorini

**Affiliations:** Assistant Features Editor, The Plant Cell, American Society of Plant Biologists; Institute of Molecular Plant Sciences, University of Edinburgh, Edinburgh EH9 3BF, UK

Glandular trichomes are epidermal outgrowths, described as “natural cell factories” because they synthesize large amounts of specialized metabolites (Huchelmann et al. 2017). Many of these metabolites play a role in protecting the plant from biotic and abiotic stresses, including extreme temperatures, high UV light, and herbivory (Wagner 1991; Huchelmann et al. 2017). Glandular trichomes are therefore an important target for crop improvement. With a developmental understanding comes the possibility of precise genome editing for breeding crops with desirable glandular trichome traits, density, and distribution (Schuurink and Tissier 2020). Cucumber (*Cucumis sativus* L.) is an economically important vegetable that is grown worldwide (Huang et al. 2009). The cucumber fruit epidermis contains multicellular glandular trichomes that synthesize, store, and deposit silicon. Silicon is a major component in fruit bloom: a fine off-white powder that forms on the cucumber fruit surface (Zhang et al. 2025). Fruit bloom can be beneficial to crop plants by reducing pathogen invasion and improving drought resistance (e.g. Ebercon et al. 1977; Samuels et al. 1993). However, due to consumer preference for bright and glossy fruits, fruit bloom has a negative effect on cucumber market value (Zhang et al. 2025).

In a recent study, Yaqi Zhang and colleagues (Zhang et al. 2025) identified key molecular components controlling bloom formation on the cucumber fruit epidermis. Two cucumber inbred lines were used: one with a fruit bloom phenotype (3548-1) and one with a non–fruit bloom phenotype (3649-1). Crossing and backcrossing experiments revealed that the non–fruit bloom phenotype resulted from a single mendelian recessive gene, and its genomic location was deduced through bulk segregation analysis sequencing and fine mapping. This genomic region contained the *CsLsi2* gene, which encodes a silicon efflux transporter. In plants with a non–fruit bloom phenotype, the *CsLsi2* gene had a single-nucleotide polymorphism that introduced a premature stop codon, making the protein nonfunctional. *CsLsi2* was highly expressed within the glandular trichomes of cucumber fruit. Cucumber CRISPR-Cas9–mediated *cslsi2* knockout mutants had non–fruit bloom phenotypes, and transmission electron microscopy–energy-dispersive spectroscopy revealed that wild type plants contained silicon in the extracellular regions of glandular trichome cell walls, which the mutants lacked. These experiments demonstrate that silicon efflux mediated by CsLsi2 is necessary for cucumber fruit bloom formation.

The MYB transcription factor CsRAX3 was identified as a potential upstream regulator of *CsLsi2* through a yeast 1-hybrid library screen. Additional biochemical assays confirmed that CsRAX3 could bind to the *CsLsi2* promoter and gene in vitro and activate transcription in planta. *CsRAX3* transcript levels were high in glandular trichomes as compared with areas of the fruit surface lacking trichomes. Cucumber CRISPR-Cas9–mediated *csrax3* knockout lines had a reduced fruit bloom phenotype and lower *CsLsi2* expression levels when compared with wild type plants. Cumulatively, this shows that CsRAX3 likely directly activates *CsLsi2* and, in doing so, promotes bloom formation.

Glandular trichome density was similar in cucumber fruits of wild type, *cslsi2*, and *csrax3* plants. Given that glandular trichomes secrete the silicon that forms fruit bloom, the authors selected a gene involved in glandular trichome development (*CsTBH*; Zhang et al. 2021) as a potential candidate acting further upstream in the regulatory pathway. A combination of biochemical assays, gene expression analyses, and investigations with *cstbh* knockout lines revealed that CsTBH likely directly regulates *CsRAX3* and *CsLsi2* (Fig.). As such, CsTBH regulates glandular trichome development and silicon deposition.

**Figure.**
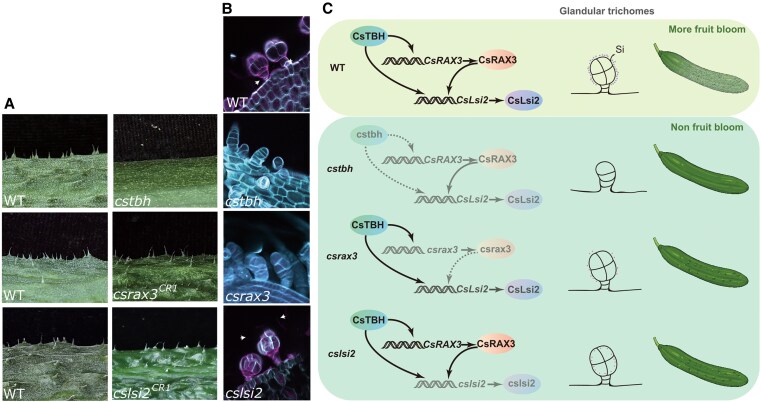
Fruit bloom formation in cucumber fruits requires silicon efflux mediated by CsLsi2. **A)** Images of the cucumber fruit epidermis from knockout mutant lines (*cstbh*, *csrax*, *cslsi2*) and corresponding wild type (WT) plants. **B)** Confocal microscopy images of *cstbh, csrax3*, *cslsi2*, and WT cucumber glandular trichomes with stained lignin and cellulose. **C)** A schematic summarizing our current understanding of how the CsTBH-CsRAX3-CsLsi2 module regulates fruit bloom formation in cucumber. Adapted from Zhang et al. (2025; Figures 2, 4, 5, 7).

Overall, Zhang et al. (2025) have provided a genetic understanding of the formation of cucumber fruit bloom. This research has potential application in breeding cucumbers with desirable fruit traits, including bloom-free varieties. The genetic regulators identified here could also be used as molecular hooks in future studies to explore the wider regulatory network and cellular mechanisms that underlie the remarkable ability of glandular trichomes to act as natural cell factories.

## Recent related articles in *The Plant Cell*

- Wu et al. (2024) show that tomato (*Solanum lycopersicum*) multicellular trichome development involves cell expansion in basal cells and cell division in apical cells. This is regulated by a gradient of a HD-Zip IV regulator (Woolly) and key downstream targets.
- Shen et al. (2024) show that *CsTL* regulates cucumber tendril formation, and they identify an interaction partner (CsTEN) and downstream target (*CsUFO*). These findings could benefit crop breeding by enabling the production of cucumber varieties lacking tendrils.

## Data Availability

No new data were generated or analysed in support of this research.

## Dive Curated Terms

The following phenotypic, genotypic, and functional terms are of significance to the work described in this paper:

glandular trichome AmiGo: PO:0004509

fruit AmiGo: PO:0009001

silicon CHEBI: CHEBI:27573
